# Effectiveness of lidocaine/prilocaine cream on cardiovascular reactions from endotracheal intubation and cough events during recovery period of older patients under general anesthesia: prospective, randomized placebo-controlled study

**DOI:** 10.1186/s12877-020-01567-y

**Published:** 2020-05-04

**Authors:** Linsheng Lv, Yan Lei, Liu Xun, Miaoxiaiven Chen

**Affiliations:** 1 Operation Room, the third Affiliated Hospital of Sun Yat-sen University, Guangzhou, 510630 Guangdong China; 2 Shanghai Shyndec Pharmaceutical Co., Ltd, Shanghai, 600420 China; 3 Division of Nephrology, the third Affiliated Hospital of Sun Yat-sen University, Guangzhou, 510630 Guangdong China; 4 Nursing Department, the third Affiliated Hospital of Sun Yat-sen University, Guangzhou, 510630 Guangdong China

## Abstract

**Background:**

Endotracheal intubation is known to pose significant physiological, pharmacokinetic, and dynamic changes and postoperative respiratory complications in patients under general anesthesia.

**Method:**

An RCT trial was organized by the Third Affiliated Hospital at Sun Yat-sen University, China. Patients were eligible for inclusion in the trial if they were over 60 years old and had upper-abdominal surgery during the induction of anesthesia and had enrolled in endotracheal intubations. The primary end point included cardiovascular reactions during the induction of anesthesia and endotracheal intubations and cough events during the recovery period. In the test group, 2 g of lidocaine/prilocaine cream (and in the control group, 2 g of Vaseline) were laid over the surface of the tracheal tube cuff.

**Results:**

The systolic blood pressure (F value = 62.271, *p* < 0.001), diastolic blood pressure (F value = 150.875, *p* < 0.001), and heart rate (F value = 75.627, *p* < 0.001) of the test group were significantly lower than the control group. Cough events during the recovery period in the test group were better (spontaneous cough, χ^2^ value = 10.591, *p* < 0.001; induced cough, χ^2^ value =10.806, *p* < 0.001).

**Conclusion:**

In older patients, coughing and cardiovascular reactions under anesthesia and endotracheal intubations were reduced, as a result of using lidocaine/prilocaine cream on the surface of the tracheal tube cuff.

**Trial registration:**

International Clinical Trials Network NCT02017392, 2013-12-16.

## Background

Endotracheal intubation has been shown to cause significant physiological, pharmacokinetic, and dynamic changes, along with postoperative respiratory complications in patients under general anesthesia [1]. Transient hemodynamic changes during these periods increase myocardial oxygen consumption, leading to myocardial ischemia. This can increase the risk of cardiovascular and cerebrovascular diseases, and of morbidity and mortality, in older patients under general anesthesia. Stellate ganglion block can restrain the stress response from inducing anesthesia and endotracheal intubations [2]. Several alternative approaches to mitigating this response have also been reported, such as the administration of esmolol, labetalol, nitroglycerin, clonidineare, and lidocaine [3–7]. However, stellate ganglion blocks are invasive and the alternative approaches are not always satisfactory.

Lidocaine/prilocaine cream (a eutectic mixture of lidocaine 25 mg/mL and prilocaine 25 mg/mL) is widely used as a topical anesthetic [8–11]. To our knowledge, however, no high quality randomized controlled study has been conducted to investigate the use of lidocaine/prilocaine cream to prevent cardiovascular stress responses in older patients undergoing tracheal intubation under general anesthesia. Our central hypothesis is that lidocaine/prilocaine can be used substantially in tracheal intubation of older patients under general anesthesia. The purpose of our study was to carry out an RCT trial on this application. The results offer accurate evidence for the local use of lidocaine/prilocaine cream in tracheal intubation of older patients under general anesthesia, to facilitate the postoperative rehabilitation and quality of life of these patients.

## Methods

### Trial design

The RCT trial was organized by the Third Affiliated Hospital at Sun Yat-sen University, China. The trial was conducted according to the CONSORT-2010 guidelines. The study was approved by the Review Board of the Third Affiliated Hospital at Sun Yet-sen University ([2013]2–85). All subjects provided written informed consent before the trial. Underlying data are available in accordance with the Management of Human Genetic Resources in China.

### Participants and setting

**Fig. 1:**
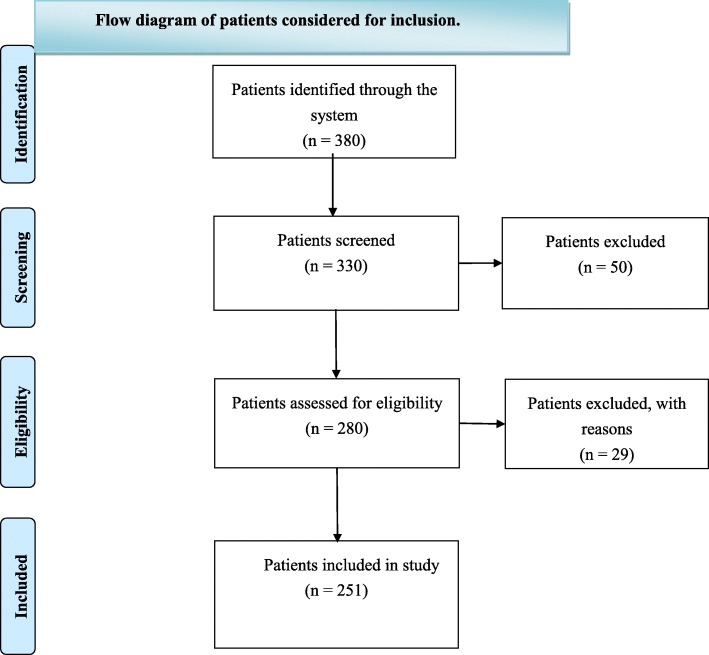
Flow diagram of patients considered for inclusion.

Patients were included in the trial if they were over 60 years old and scheduled for upper abdominal surgery under general anesthesia using endotracheal tube intubation from August 2015 through December 2018. The major exclusion criteria were the following: allergies to lidocaine, prilocaine, or any other ingredients in the test product; ischemic heart disease or advanced chronic kidney disease; younger than 60 years old; American Society of Anesthesiologists (ASA) Grade IV. We randomly assigned older patients to either lidocaine/prilocaine cream treatment or a placebo (Vaseline) at a 1:1 ratio. The primary end point included cardiovascular reactions during the induction of anesthesia, along with endotracheal intubations and cough events during the recovery period. Figure 1 shows a flowchart for the assignment of participants in the study.

### Intervention

Nurses inflated the endotracheal tube. According to the random number table, in the test group, off-label use of 2 g of lidocaine/prilocaine cream (and in the control group, 2 g of Vaseline cream) were laid over the surface of the tracheal tube cuff. After ventilation, the anesthesiologists placed the endotracheal tube and fixed the endotracheal tube with air. Both the patients and the investigators were unaware of the trial-group assignments. Before tracheal intubation, anesthetics was used and unconscious intubation was carried out. The depth of anesthesia was measured by the bispectral index (BIS). Fentanyl was used as the induction agent according to the sex, weight, and ASA rating of the patients. Benzene sulfuro acted as the muscle relaxant and its dosage was based on the weight and situation of the patients during the operation.

### Parameter measurement

Cardiovascular reaction during the induction of anesthesia: systolic blood pressure (SBP) (mm Hg), diastolic blood pressure (DBP) (mm Hg), heart rate (HR) (beats/min), and cough reaction. Coughing that occurred during extubation was defined as induced cough. The SBP, DBP, and HR were measured with a monitor.

### Sample size calculation

According to the pretest analysis of 10 patients in each group, we set α = 0.05 and β = 0.20, with a sample drop rate of 20%. Using Open Epi Version 2 [12], we calculated the minimum sample size of 125 cases in each group (a total of 250 cases).

### Statistical analysis

The measurements were calculated as the mean (± standard deviation). An independent *t*-test was used to compare the groups. Further, a chi-squared test was used to compare the categorized data in the groups. An analysis of variance of the repeated measurement data was conducted to compare the data. We set *p* < 0.05 as statistically significant. R version 3.0.2 was used to process the data.

## Results

### Patients

A total of 251 older patients at the Third Affiliated Hospital at Sun Yet-sen University were enrolled in the randomized trial. There were no significant differences in the baseline characteristics (such as age, sex, ASA rating, operation time and depth of anesthesia) of the patients between the two groups (Table 1). Both groups were anesthetized with general anesthesia by tracheal intubation.

**Table 1:** Patient Characteristics

	Test Group	Control Group	t/χ^2^ Value	*P* Value
Age (years)	70.3 ± 5.9	70.4 ± 5.2	−0.155	0.877
Sex (male n (%))	76 (53.9)	65 (46.1)	2.163	0.141
ASA I (%)	44 (51.8)	41 (48.2)	0.752	0.687
ASA II(%)	38 (52.1)	35 (47.9)		
ASA III(%)	43 (46.2)	50 (53.8)		
Operation time (hours)	1.6 ± 0.6	1.6 ± 0.6	t = −0.693	0.489
Depth of anaesthesia	48.3 ± 4.4	47.6 ± 4.5	t = 1.279	0.202

### Primary outcomes

The systolic blood pressure (F value = 62.271, *p* < 0.001), DBP (F value = 150.875, *P* < 0.001) and heart rate (F value = 75.627, *P* = < 0.001) of the test group were significantly lower than the control group (Table 2).

**Table 2:** Comparison of cardiovascular reaction and heart rate between two groups

	Group	Before induction	Before intubation	Instant intubation	1 min after intubation	Instant extubation	1 min after extubation	3 min after extubation	F Value	*P* Value
SBP (mm Hg)	Test group	141.8 ± 5.9	131.8 ± 5.3	128.8 ± 4.8	126.8 ± 4.6	123.8 ± 3.9	120.8 ± 3.7	119.9 ± 3.4	62.271	< 0.0001
	Control group	139.7 ± 5.4	133.8 ± 4.9	131.8 ± 4.6	130.8 ± 4.2	131.9 ± 3.7	129.8 ± 3.5	127.9 ± 3.3		
DBP (mm Hg)	Test group	81.8 ± 5.9	74.8 ± 5.3	73.8 ± 4.8	74.8 ± 4.6	69.8 ± 3.9	71.8 ± 3.7	69.9 ± 3.4	150.875	< 0.0001
	Control group	82.7 ± 5.4	75.8 ± 4.9	81.8 ± 4.6	80.8 ± 4.2	82.9 ± 3.7	80.8 ± 3.5	79.9 ± 3.3		
HR (beat/min)	Test group	85.8 ± 5.9	77.8 ± 5.3	82.8 ± 4.8	80.8 ± 4.6	82.8 ± 3.9	80.8 ± 3.7	82.9 ± 3.4	75.627	< 0.0001
	Control group	85.7 ± 5.4	77.8 ± 4.9	89.8 ± 4.6	88.8 ± 4.2	89.9 ± 3.7	88.8 ± 3.5	86.9 ± 3.3		

^a^SBP: systolic blood pressure; DBP: diastolic blood pressure; HR: heart rate; min: minute

Cough events in recovery period in the test group were better (Spontaneous cough (χ^2^ value = 10.591, *P* = 0.001); Induces cough (χ^2^ value =10.806, *P* = 0.001)) (Table 3).

**Table 3:** Comparison of cough events during recovery period between two groups

	Treatment Group	Control Group	χ^2^ Value	*P* Value
Spontaneous cough [case (%)]	4 (3.1)	13 (10.7)	10.591	0.001
Induces cough [case (%)]	8 (6.4)	27 (21.4)	10.806	0.001

## Discussion

To our knowledge, this study for the first time demonstrated in a high quality RCT that there are improvements from the use of lidocaine/prilocaine cream on the surface of the tracheal tube cuff in older patients in terms of postoperative coughing and cardiovascular reactions during the induction of anesthesia. Our results are similar to those by Chen [2]. Furthermore, our study found that cough events during the recovery period improved in the test group. These findings may lead to beneficial effects on cardiovascular reactions in the course of endotracheal intubation.

During tracheal intubation, blood pressure often rises sharply, and systolic blood pressure rises by 45 mmHg on average. Further, tachycardia and other circulatory system reactions are common, and they are collectively referred to as the intubation stress response. Generally, the time is short (3–5 min). However, patients with an abnormal cardiovascular and cerebrovascular system, especially older patients, face life-threatening reactions that should be minimized or avoided completely [13]. Lidocaine and prilocaine are phthalocaine local anesthetics. Lidocaine is fast-acting, with wide dispersion, strong penetration, and no obvious vasodilator effect. The structure of prilocaine is similar to that of lidocaine and it decomposes easily, such that its toxicity is relatively rare. Its onset time is slower than that of lidocaine, and its duration is slightly longer. The combined application of the two drugs can enhance the anesthetic effect. Moreover, it takes effect quickly with a long duration. It has antimicrobial properties of intact human skin flora as a topical anesthesia before vascular access, and reduces the pain of venipuncture in hemodialysis patients [8–11].

The strength of our paper lies in its high-quality design. However, there are limitations: 1) the total observation period was relatively short, and indeed a longer observation time may be better; 2) although our treatment was very safe, we did not extensively analyze side effects; 3) we did not set up clinically relevant cutoffs regarding blood pressure; 4) we did not study significant postoperative symptoms such as postoperative sore throat or difficulty swallowing in this paper; and 5) the coughing intensity was not measured.

## Conclusion

We found that during the induction of anesthesia in older patients, cough reactions and cardiovascular reactions from endotracheal intubations improved as a result of using lidocaine/prilocaine cream on the surface of the tracheal tube cuff, which may decrease the risk of cardiovascular and cerebrovascular diseases in these patients.

## Data Availability

The database used and/or analyzed during the current study are available from the corresponding author (Prof. Xun Liu, Division of Nephrology, the third Affiliated Hospital of Sun Yat-sen University, Guangzhou 510630, China. Tel: + 86 020 8525 3115, e-mail: naturestyle@163.com) on reasonable request.

## Abbreviations

RCT: Randomized controlled study
SBP: Systolic blood pressure
DBP: Diastolic blood pressure
HR: Heart rate
