# Correction to: Effectiveness of lidocaine/prilocaine cream on cardiovascular reactions from endotracheal intubation and cough events during recovery period of older patients under general anesthesia: prospective, randomized placebo-controlled study

**DOI:** 10.1186/s12877-020-01651-3

**Published:** 2020-09-11

**Authors:** Linsheng Lv, Lei Yan, Xun Liu, Miaoxia Chen

**Affiliations:** 1 Operation Room, the third Affiliated Hospital of Sun Yat-sen University, Guangzhou, 510630 Guangdong China; 2 Shanghai Shyndec Pharmaceutical Co., Ltd, Shanghai, 600420 China; 3 Division of Nephrology, the third Affiliated Hospital of Sun Yat-sen University, Guangzhou, 510630 Guangdong China; 4 Nursing Department, the third Affiliated Hospital of Sun Yat-sen University, Guangzhou, 510630 Guangdong China

**Correction to: BMC Geriatr 20, 157 (2020)**

**https://doi.org/10.1186/s12877-020-01567-y**

Following publication of the original article [1], we have been notified that there is a mistake in authors’ first and last names.

Currently authors first and last names are: Linsheng Lv, Yan Lei, Liu Xun and Miaoxiaiven Chen.

They should be as follows: Linsheng Lv, Lei Yan, Xun Liu and Miaoxia Chen.

The original article [1] has been corrected.
